# Frequency of depression and correlates among Chinese children and adolescents living in poor areas under the background of targeted poverty alleviation: results of a survey in Weining county

**DOI:** 10.1186/s12888-023-05334-2

**Published:** 2023-11-08

**Authors:** Xiaofei Yuan, Tingting Hu, Xiaorui Zhu, Sixin Dong, Gang Wang, Xu Chen, Jiaojiao Zhou

**Affiliations:** 1 The National Clinical Research Center for Mental Disorders & Beijing Key Laboratory of Mental Disorders, Beijing Anding Hospital & the Advanced Innovation Center for Human Brain Protection, Capital Medical University, No. 5 Ankang Hutong, Xicheng District, Beijing, 100035 China; 2 Beijing Municipal Administration of Hospitals, Beijing, China

**Keywords:** Adolescent, Depression, Factors, Poverty, Internet addiction

## Abstract

**Background:**

There is a lack of epidemiological data on depressive morbidity in children and adolescents in rural China. This study determines the frequency and correlates of depression among children and adolescents to offer useful insights for family education and government policy-making in rural China.

**Methods:**

A cross-sectional online survey was conducted between April 20 to May 10, 2022. Depression was assessed using the Center for Epidemiologic Studies Depression Scale (CES-D), and the correlative factors of depression were analyzed.

**Results:**

In this study, 23,180 children and adolescents were enrolled (median (range) age: 12 (9–18) years); of them, 8,261 (35.6%) suffered from depression with a CES-D score of > 15. The onset of depression was significantly related to age, grade, gender, parental absence, attending key schools or classes, presence of moderate or severe internet addiction (IA), school record, social relationships, parental occupation, and education status. Furthermore, female gender (OR = 1.175; 95% CI: 1.108–1.247; p < 0.001), junior middle school (OR = 1.487; 95% CI: 1.380–1.601; p < 0.001), parental absence (OR = 1.272; 95% CI: 1.183–1.367; p < 0.001), attending key schools (OR = 1.221; 95% CI: 1.120–1.332; p < 0.001), attending key classes (OR = 1.099; 95% CI: 1.001–1.207; p = 0.048), and presence of moderate or above IA (OR = 13.593; 95% CI: 12.028–15.361; p < 0.001) were the most prominent independent factors for depression.

**Conclusion:**

Depression is very common among Chinese children and adolescents living in poor areas of Weining County. Older age, higher school grade, female gender, parental absence, attending key schools or classes, and the presence of moderate to severe IA are some important factors that may dictate the occurrence of depression in these children and adolescents.

## Introduction

Depression is a common mental disorder with grave affective, cognitive, and psychomotor sequelae among adolescents [1, 2]. A meta-analysis of 51 studies, involving 144,060 adolescents from mainland China, reported an estimated the depression prevalence of 24.3% [3]. Depression in children and adolescents may result in substance abuse, diminished educational achievement, delinquency, and even suicide—one of the leading causes of adolescent mortality [4]. Besides, adolescent-onset mental disorders often persist through adulthood and may perpetuate into future generations [5]. Thus, strategies aimed at early prevention, detection, and treatment are extremely crucial to protect children and adolescents from depression.

Over the past three decades, the Chinese government has implemented a battery of actions to eliminate poverty and promote the health of rural and economically underprivileged residents. The targeted poverty alleviation (TPA) policy implemented in 2016 by the Chinese government has been a great influence assuaging poverty among rural poor residents by promoting income, health, and living standards through comprehensive poverty reduction strategies and building a moderately well-rounded prosperous society [6]. Hitherto, several studies have diligently evaluated the TPA policies and China’s rural medical effects and policies; however, there is a dearth of literature addressing the health status, especially mental health, of the rural poor citizens in the backdrop of the TPA policy [6, 7].

It has been reported that poverty is associated with disabilities and illness, which has long been a great concern for the Chinese government [8]. Furthermore, the current state of mental health associated with socioeconomic inequality is becoming a serious public health concern in China [9], such as the prevalence of depressive disorders varying greatly among different socioeconomic classes [10, 11]. People belonging to the lower-income strata tend to have a higher incidence of depression [10–12]. An equality-related study reported that the depressive symptoms in elderly Chinese people tend to occur more in the lower-income groups [13]. However, the prevalence of depression among Chinese children and adolescents belonging to lower-income groups remains unclear.

Notably, children and adolescents in rural China often suffer from parental absence; according to the Ministry of Civil Affairs, China, there were approximately 6,436 million rural left-behind children in China in 2020 alone [14]. Parental absence not only predisposes adolescents to unwarranted economic and social disadvantages but may also affect their general well-being and psychological health [14]. Bowlby’s attachment theory suggests that children who experience long-term separation from their attached person, tend to suffer from emotional distress, such as anger, anxiety, and depression [15]. Therefore, prompt actions are needed to strengthen the prevention, detection, and management of depression in vulnerable groups. However, there is a lack of epidemiological data on depressive morbidity in children and adolescents in rural China. Therefore, we conducted a large cross-sectional sample survey to determine the prevalence and correlates of depressive symptoms among children and adolescents in Weining, a county-level city located in Southwest China in Guizhou Province, to offer useful insights for family education and government policy-making in rural China.

## Methods

### Study design and participants

A cross-sectional online survey was conducted between April 20 to May 10, 2022, based on the collaborative research network of the National Clinical Research Center for Mental Disorders, China. The survey was conducted by snowball sampling in Weining County using the WeChat-based Wenjuanxing program (https://www.wjx.cn/). In our study, the existing subjects provide referrals to recruit samples required. WeChat is widely used for student management in most schools in China. School students aged 9–18 years living in the Weining Country were included in the study. Participants who completed the questionnaire in < 120 s or were outside the 9–18 years age group were excluded. All students completed the questionnaire under the guidance of teachers or guardians.

### Assessment instruments and data collection

A data collection sheet designed for this study was used to collect socio-demographic and clinical characteristics, such as age, gender, school type, grade and class type, school record, parental education level, parental occupation, parental absence (absence of at least one parent), family relationship, classmate relationship, teacher-student relationship, and presence of network addiction and depression. Key schools are those that are supported by the local government, have better basic teaching facilities and teachers, and have higher requirements for students (The key schools in each area are well known). Key classes refer to those with good academic performance and strong teachers. Social relationships and school record were classified according to children and adolescents’ self-satisfaction. Well-educated was defined as college degree or above. Well occupation was defined as one that has a steady income and is formal, such as teachers, medical staff, civil servants, or self-employed people with good incomes.

The presence of depression was assessed by the Center for Epidemiological Studies Depression Scale (CES-D), Chinese version [16], which was developed for use in surveys of depressive symptomatology in the general population and was a short, structured self-report scale [17]. The CES-D scale was proved to be a reliable and measure valid for use with adolescents [18]. Total scores range from 0 to 60, and a CES-D cut-off score of 16 had sensitivity of 100% and specificity of 76% [19]. The presence of internet addiction (IA) was assessed by Young’s Internet Addiction Test (IAT), which was composed of 20 items with a total score ranging from 20 to 100. And higher scores reflect a greater tendency toward IA (0–30: no IA; 31–49: mild level of IA; 50–79: moderate level of IA; 80–100: severe level of IA) [20].

### Statistical analyses

The Pearson’s and Chi-squared tests were used to determine the distribution of categorical variables, while the Mann-Whitney U test was used for continuous variables. Univariate and multivariate analyses were performed to examine the correlates of depression using the logistic regression model. When multiple factors w closely related, such as age and grade type, only one of them was included in the multivariate analysis. The enter method was adopted for multivariable logistical regression analyses. All statistical tests were performed using SPSS version 22.0 (IBM Corp, Armonk, NY, USA); statistical significance was determined using a p-value of < 0.05 (two-tailed).

## Results

### Sample characteristics and prevalence of depression

A total of 23,596 children and adolescents were invited to participate in the survey, of which 416 were excluded—106 completed the questionnaire in less than 120 s, and 310 did not belong to the 9–18 years age group. Finally, 23,180 children and adolescents were enrolled (median (range) age: 12 (9–18) years); of them, 8,261 (35.6%) suffered from depression with a CES-D score of > 15. The socio-demographic and clinical characteristics of both the no-depression and depression groups are summarized in Table 1. Depressive symptoms were more common among adolescents aged 12–18 years old (37.4% vs. 33.3%, p < 0.001), junior middle school (46.3.% vs. 32.5%, p < 0.001), females (36.4% vs. 34.9%, p = 0.013), suffering from parental absence (42.4% vs. 33.9%, p < 0.001), attending key schools (41.2% vs. 34.6%, p < 0.001), attending key classes (86.3% vs. 29.8%, p < 0.001), and those suffering from moderate or above IA (46.4% vs. 32.7% vs. 34.4%, p < 0.001). On the other hand, adolescents who were living with well-educated parents (31.0% vs. 36.0%, p < 0.001), having sound parental occupations (30.3% vs. 36.6%, p < 0.001), good school record (24.6% vs. 38.8%, p < 0.001), or good social relationships (family, student and teacher–student relationship, all p < 0.001) tended to have a lower rate of depression.

**Table 1:** Demographic characteristics of the study sample (n = 23,180)

Variables	Total, n (%)	Depression		p value
		No, n (%)	Yes, n (%)	
Age, year,‾x ± s	12.14 ± 2.22	11.96 ± 2.07	12.46 ± 2.43	< 0.001*
Gender				0.013*
Female	11,404 (49.2)	7249 (63.6)	4155 (36.4)	
Male	11,776 (50.8)	7670 (65.1)	4106 (34.9)	
Grade				< 0.001*
Primary school	17,965 (77.5)	12,120 (67.5)	5846 (32.5)	
Secondary school	5215 (22.5)	2799 (53.7)	2416 (46.3)	
Well-educated parents				< 0.001*
No	20,880 (90.1)	13,333 (63.9)	7547 (36.1)	
Yes	2300 (9.9)	1586 (69.0)	714 (31.0)	
Well parental occupation				< 0.001*
No	19,618 (84.6)	12,436 (63.4)	7182 (36.6)	
Yes	3562 (15.4)	2483 (69.7)	1079 (30.3)	
Parental absence				< 0.001*
No	18,500 (79.8)	12,223 (66.1)	6277 (33.9)	
Yes	4680 (20.2)	2696 (57.6)	1984 (42.4)	
Attending key schools				< 0.001*
No	19,548 (84.3)	12,784 (65.4)	6764 (34.6)	
Yes	3632 (15.7)	2135 (58.8)	1497 (41.2)	
Attending key classes				< 0.001*
No	19,766 (85.3)	12,988 (65.7)	6778 (34.3)	
Yes	3413 (14.7)	1931 (56.6)	1482 (43.4)	
Good school record				< 0.001*
No	18,074 (78.0)	11,067 (61.2)	7007 (38.8)	
Yes	5106 (22.0)	3852 (75.4)	1254 (24.6)	
Moderate or above IA^#^				< 0.001*
No	20,786 (89.7)	14,592 (70.2)	6194 (29.8)	
Yes	2394 (10.3)	327 (13.7)	2067 (86.3)	
Good family relationship				< 0.001*
No	1713 (7.4)	712 (41.6)	1001 (58.4)	
Yes	21,467 (92.4)	14,207 (66.2)	7260 (33.8)	
Good student relationship				< 0.001*
No	1643 (7.1)	689 (41.9)	954 (58.1)	
Yes	21,537 (92.9)	14,230 (66.1)	7307 (33.9)	
Good teacher-student relationship				< 0.001*
No	1520 (6.6)	696 (45.8)	824 (54.2)	
Yes	21,660 (93.4)	14,223 (65.7)	7437 (34.3)	

# IA: internet addiction

* Statistically significant

### Characteristics associated with depression

The univariate logistic regression analysis showed that participants with the following characteristics had higher odds of suffering from depression (Table 2): aged 12–18 years (odds ratio, OR = 1.194; 95% confidence intervals, CI: 1.131–1.261; p < 0.001), females (OR = 1.071; 95% CI: 1.015–1.130; p = 0.013), were in junior middle school (OR = 1.790; 95% CI: 1.681–1.906; p < 0.001), suffering from parental absence (OR = 1.790; 95% CI: 1.681–1.906; p < 0.001), attended key schools (OR = 1.325; 95% CI: 1.233–1.425; p < 0.001), attended key classes (OR = 1.325; 95% CI: 1.233–1.425; p < 0.001), and suffered from moderate or above IA (OR = 14.891; 95% CI: 13.203–16.796; p < 0.001). Contrarily, adolescents who were with well-educated parents (OR = 0.795; 95% CI: 0.725–0.873; p < 0.001), had sound parental occupations (OR = 0.752; 95% CI: 0.697–0.813; p < 0.001), good school record (OR = 0.514; 95% CI: 0.479–0.552; p < 0.001), and good social relationships (family, student and teacher–student relationship, all OR < 1 and p < 0.001) were less likely to develop depression (Table 2).

**Table 2:** Univariate and multivariable logistical regression analyses of correlates of depression※

Variables	Univariate analyses		Multivariate analyses	
	OR(95%CI)	p value	OR(95%CI)	p value
Gender, female	1.071 (1.015–1.130)	0.013*	1.175 (1.108–1.247)	< 0.001*
Grade, Secondary school	1.790 (1.681–1.906)	< 0.001*	1.487 (1.380–1.601)	< 0.001*
Well-educated parents	0.795 (0.725–0.873)	< 0.001*	1.042 (0.926–1.172)	0.498
Well parental occupation	0.752 (0.697–0.813)	< 0.001*	0.838 (0.760–0.925)	< 0.001*
Parental absence	1.433 (1.342–1.530)	< 0.001*	1.272 (1.183–1.367)	< 0.001*
Attending key schools	1.325 (1.233–1.425)	< 0.001*	1.221 (1.120–1.332)	< 0.001*
Attending key classes	1.471 (1.366–1.583)	< 0.001*	1.099 (1.001–1.207)	0.048*
Good school record	0.514 (0.479–0.552)	< 0.001*	0.540 (0.500-0.584)	< 0.001*
moderate or above IA^#^	14.891 (13.203–16.796)	< 0.001*	13.593 (12.028–15.361)	< 0.001*
Good family relationship	0.363 (0.329–0.402)	< 0.001*	0.544 (0.464–0.637)	< 0.001*
Good student relationship	0.371 (0.335–0.411)	< 0.001*	0.574 (0.487–0.678)	< 0.001*
Good teacher-student relationship	0.442 (0.398–0.490)	< 0.001*	1.154 (0.971–1.371)	0.104

# IA: internet addiction

* Statistically significant

※The enter method is adopted for multivariable logistical regression analyses and all independent variables were entered at the same time

Using the multivariable logistic regression model, we found that the female gender (OR = 1.175; 95% CI: 1.108–1.247; p < 0.001), junior middle school (OR = 1.487; 95% CI: 1.380–1.601; p < 0.001), parental absence (OR = 1.272; 95% CI: 1.183–1.367; p < 0.001), attending key schools (OR = 1.221; 95% CI: 1.120–1.332; p < 0.001), attending key classes (OR = 1.099; 95% CI: 1.001–1.207; p = 0.048), and presence of moderate or above IA (OR = 13.593; 95% CI: 12.028–15.361; p < 0.001) were the most prominent independent factors for depression (Table 2).

## Discussion

It is known that the prevalence of depression is both directly and indirectly affected by the population’s socioeconomic status, and people with relatively lower incomes have a higher incidence of depression [10, 12]. Poverty has a significant negative impact on the daily living and education of adolescents, especially those experiencing parental absence or living in remote areas, resulting in a high risk of depression. As a developing country with one-fifth of the world population, China has millions of children and adolescents living in poverty. However, the frequency of depression and its correlates in Chinese children and adolescents living in economically backward areas has not well been addressed [6, 7]. In this cross-sectional survey, we found that 35.6% of Chinese school-going children and adolescents (9–18 years) living in poor areas suffered from depression despite the existence of the TPA policy. The depressive symptoms were significantly related to multiple factors including the participant’s age, grade, gender, parental absence, attending key schools or classes, presence of moderate or severe IA, school record, social relationships, parental occupation, and education status. Appropriate knowledge of these factors may be enlightening for stakeholders to undertake immediate actions for strengthening the prevention, detection, and management of depression in high-risk groups.

A meta-analysis and systematic review of 144,060 Chinese adolescents reported a pooled prevalence of depression of 24.3% (95% CI: 21.3–27.6) [3]. We observed a rather alarmingly higher incidence of depression in our study sample, which indicates that, to some extent, poverty is an important risk factor for depression in children and adolescents. Previous studies indicated that lower socioeconomic status tends to reduce people’s enthusiasm to invest in health, and residents with lower socioeconomic status face greater health risks, especially higher depression risk [6, 12, 21]. In this regard, child poverty has detrimental effects, more so on the child’s mental health [22]. Lower family income during childhood has been proved to be related to an increased incidence of depression among adolescents, although the potential mechanisms remain unclear [23]. A recent study reported that lower neuronal signaling in the prefrontal cortex is related to childhood family income and depressive symptomatology during adolescence [24]. Future studies must attempt at elucidating the mechanisms underlying the negative relation between poverty and depression.

Our results also indicated that female adolescents aged 12–18 years old had a higher risk of depression, which was consistent with previous studies [25, 26]. It has been often reported that females have a higher prevalence of depressive symptoms than males during adolescence, which was one of the best-replicated findings in the study focused on depression [27]. The change in the incidence of depression tends to occur at the same time as the physical and hormonal changes of puberty because many behavioral, psychological, and social transitions accompany the physiological changes [28]. In our study, children and adolescents with higher grades and those attending key schools or classes were more likely to suffer from depression, which was comparable to the existing literature [28, 29]. In China, children and adolescents in higher grades and attending key schools or classes often face greater academic stress of sitting various college entrance examinations [30] which may have resulted in a higher incidence of depression in this group.

Consistent with previous studies, we also observed that children and adolescents separated from their parents tended to have depression [15, 31]. Parental absence often renders adolescents more likely to be exposed to danger, resulting in emotional issues and externalized behavioral risks [32]. Additionally, adolescents with parental distance tend to have more negative emotions, such as guilt, despair, loneliness, and despair [33, 34], besides having lower psychological resilience [35], lower self-esteem [36], and poorer interpersonal relationships [37]. In our study, adolescents who had better interpersonal relationships were less likely to suffer from depression.

Furthermore, we found that children and adolescents with moderate or above IA had a higher risk of depression. Previous studies indicated that high rates of depression could be observed in individuals with IA [38, 39]. IA is also associated with poor sleep quality, low self-esteem, and lower levels of physical activity [40], which often lead to depression. Contrarily, we found that children and adolescents with well-educated parents or sound parental occupations were at a lower risk of depression.

The present study had certain limitations. First, the study design originally screened out the more deprived segment of the population that did not have access to the Internet. Second, some information on important factors related to depression, such as physical health and social support, were not available due to some reasons. And we could not conclude from the results of the online questionnaire that the patient is indeed depressed and not just depressed for a short period of time. Third, causality between variables could not be established because of the cross-sectional nature of the study. Meanwhile, using snowball sampling might have influenced the sample’s representation, resulting in sampling bias. And we could not compare the difference in depression prevalence before and after the implementation of TPA. Lastly, when a threshold of 16 is used to assess depression in youth, especially in children aged 9 to 11 years, the potential bias related to false positives should be concerned. The reasonableness of using this cut-off value in children and adolescents needs to be further studied. A longitudinal study is necessary to complement epidemiological studies and provide more conclusive evidence on the prevalence and correlates of depression among economically-disadvantaged Chinese children and adolescents.

## Conclusion

Depression is very common among Chinese adolescents living in poor areas of Weining County. Older age, higher school grade, female gender, parental absence, attending key schools or classes, and the presence of moderate to severe IA are some important factors that may dictate the occurrence of depression in these adolescents.

## Data Availability

The data used in this study are available from the corresponding author upon reasonable request.

## Abbreviations

CES-D: the Center for Epidemiologic Studies Depression Scale
IA: internet addiction
TPA: targeted poverty alleviation
